# Negative Affect, Fatalism, and Perceived Institutional Betrayal in Times of the Coronavirus Pandemic: A Cross-Cultural Investigation of Control Beliefs

**DOI:** 10.3389/fpsyt.2020.589914

**Published:** 2020-10-26

**Authors:** Rahel Bachem, Noga Tsur, Yafit Levin, Hisham Abu-Raiya, Andreas Maercker

**Affiliations:** ^1^Psychopathology and Clinical Intervention, Department of Psychology, University of Zurich, Zurich, Switzerland; ^2^Bob Shapell School of Social Work, Tel-Aviv University, Tel-Aviv, Israel

**Keywords:** COVID-19, pandemic, negative affect, fatalism, locus of control, institutional betrayal, cross-cultural

## Abstract

**Background:** A growing number of studies report that the COVID-19 pandemic has resulted in diverse aversive psychological reactions and created a global mental health crisis. However, the specific mechanisms underlying the negative emotional reactions as well as the differences between countries are only beginning to be explored. The present study examined the association of COVID-19-related fear and negative affect in Israel and Switzerland. The mediating roles of three control beliefs were explored, namely, fatalism, locus of control, and perceived institutional betrayal.

**Method:** General population samples of 595 Swiss and 639 Israeli participants were recruited and completed an online self-report survey. Moderated Mediation using multigroup path analysis models for the two samples were conducted and compared using AMOS.

**Results:** The multigroup path model had excellent fit for both samples. The different paths were moderated by country affiliation. Higher levels of COVID-19-related fear were associated with negative affect to an equal extent in both samples. COVID-19-related fear was associated with higher reports of institutional betrayal and a lower locus of control in both samples. Higher COVID-19-related fear was associated with lower fatalism in the Swiss sample only. In both samples, institutional betrayal mediated the association between COVID-19-related fear and negative affect, however, locus of control was a mediator in the Israeli sample only.

**Conclusion:** The current results suggest that the reaction of the government was of crucial importance with regard to the emotional state of the two populations. Interestingly, while in the context of adversity fatalism is generally considered a risk factor for mental health, during the time of the pandemic it seems to have had protective qualities among the Swiss population. Interventions that strengthen the personal locus of control have the potential to mitigate the negative affect in Israel but not in Switzerland. Despite the fact that COVID-19 is a global phenomenon, prevention and intervention strategies should be adjusted to local contexts.

## Introduction

Since the beginning of the year 2020 the lives of people around the globe have been dominated by one particular stressor: the outbreak of the Coronavirus disease (COVID-19). A growing number of studies have reported on the extensive negative psychological reactions to COVID-19, which amount to a global mental health crisis (1). Due to the extremely high infection rates and relatively high mortality rates, the primary reactions to the pandemic have been fear, worry, and anxiety among people worldwide [e.g., (2–4)]. Emerging initial findings in a number of countries have documented evidence that high levels of COVID-19 related fear correlated substantially with elevated depression (2, 5), stress (6) poorer sleep quality (7), and lower mental wellbeing (8) in the general population. Moreover, levels of anxiety, depression and posttraumatic stress symptoms were reported to be higher during the COVID-19 pandemic than in previous population studies (9) (Shevlin et al. unpublished manuscript)^1^.

However, the specific mechanisms that determine the debilitating effect of COVID-19 on mood and emotionality as well as cultural differences in this respect, are only beginning to be explored. Additionally, research is needed to better understand the importance of the local context for the response to the outbreak. The present study, therefore, examined the association of COVID-19 related fear and negative affect in two samples collected in Israel and Switzerland with the aim to identify potential mechanisms underlying this association.

As has been observed during previous epidemics, the COVID-19 pandemic has caused global anxiety and heightened stress (3). Ornell et al. (10) pointed out that during epidemics, the number of individuals whose mental health is affected is generally higher than the number of people who suffer from the infection, which necessitates exploring current and future mental health concerns (11). The pandemic does not only foster a concrete fear of death but is also accompanied by unprecedented economic and social repercussions that affect various spheres of family structures and professional life in unpredictable manners (10). Insecurity and fear of the unknown raise anxiety levels in healthy individuals as well as those with preexisting mental health conditions (12). In China, for example, approximately half of the respondents in a general population survey reported the psychological impact of the epidemic as moderate to severe (13), whereas in Italy, 41.8% of respondents form the general population reported high distress and 37.19% indicated high levels of anxiety (14). Furthermore, uncertainty about the risk of infecting family and friends tends to potentiate dysphoric mental states (15).

Although findings have shown that most people report a certain level of COVID-19 related fear (3), the reactions to the pandemic vary widely between individuals; some develop psychopathologies while others succeed to maintain psychological balance and adapt to the situation. It is therefore important to better understand the conditions under which individuals are able to cope with the uncertainty and anxiety related to the pandemic. To date, most studies that investigated psychological responses to COVID-19 focused on sociodemographic risk factors such as gender, age, occupation, or education level [e.g., (6) (Shevlin et al. unpublished manuscript)^1^] or social variables such as social support or loneliness (16, 17). Nevertheless, other factors have not yet been thoroughly explored.

Expert opinions predominantly highlight the importance of individual control, beliefs, and perceptions of helplessness with regard to suffering from emotional distress during the present pandemic (8, 10, 18, 19). More specifically, Satici et al. (8) found that intolerance for uncertainty had a significant direct effect on mental wellbeing during the COVID-19 situation. Independent of the specific context of the COVID-19 pandemic, research has firmly suggested that self-mastery is a crucial criterion for promoting wellbeing in times of crisis (20). The current study is among the first to empirically assess individuals' unique perceptions of control on three levels: fatalistic world views (reflecting the propensity to believe that one's destiny is externally determined), health locus of control (reflecting trust in self as able to cope with the pandemic), and perceptions of institutional betrayal (reflecting trust in authorities to protect against the virus) in the context of coping with COVID-19.

The first concept of interest, fatalism, describes the general belief that one's destiny is externally determined and that one's actions have little or no significant impact on important outcomes (21). A fatalistic attitude of life can result in reduced fear and anxiety in highly threatening situations, particularly when efforts to engage in direct means of resolving the conflict seem futile (22). Thus, choosing to disengage with the stressor can be an effective way to eliminate the tension created by a situation that is perceived as threatening and uncontrollable. At the same time, however, higher fatalism has also been shown to be strongly and positively associated with hopelessness and depression [e.g., (23)] and, to a lesser extent, with increased symptoms of general psychological distress (24). Hence, in the current COVID-19 crisis, fatalistic views may have a complex effect on mental health and wellbeing. While fatalistic control beliefs may reduce COVID-19 related fears, this strategy may come at the cost of higher levels of negative affect (22).

The second concept of interest, health-related internal locus of control, refers to people's attribution of their own health to either personal or environmental factors (25). Perceived control over outcomes primes individuals to view difficult situations as challenges rather than insurmountable obstacles and enables them to choose adaptive coping strategies (26). There is an extensive body of research linking a high locus of control with psychological health, indicated by fewer symptoms of anxiety, depression, and faster recovery after confronting adverse life circumstances (27–29). In the current study, the health locus of control reflects the degree to which an individual trusts in themselves as capable of coping with the COVID-19 pandemic. A recent study conducted during the COVID-19 crisis showed that a general sense of control mediated the association between stress symptoms and positive mental health. This suggests that a sense of control fosters calmer management of the current challenges and has the potential to buffer any negative mental health consequences of the pandemic (30).

When facing a global crisis, such as the spread of COVID-19, it seems that the government and healthcare systems play a significant role in the degree to which the new virus threatens individuals and societies. Perceived institutional betrayal, the third concept of interest, occurs when people perceive powerful and trusted institutions as causing harm to those dependent on them for safety and wellbeing, either by action or inaction in times of crisis or when mistakes or crimes have been committed (31). This type of perceived betrayal has largely been discussed in the context of cover-up attempts of sexual assaults in the Catholic church, the military, or within universities (32). In addition, it is known to exacerbate various psychopathological reactions to adversity and trauma, such as anxiety, depression, and posttraumatic stress disorder (33, 34). In the context of the COVID-19 crisis, the subject of institutional betrayal is now beginning to be discussed in regards to medical systems, both because of a lack of adequate provision of care for patients as well as the failure to provide sufficient personal protective equipment to health care staff (35, 36). In the current project, perceived institutional betrayal refers to people's lack of trust in the local government and healthcare institutions to protect against the virus (i.e., the level to which participants felt that these institutions took inadequate action to protect personal and public health and wellbeing).

Despite the fact that COVID-19 is a global phenomenon, relatively few studies have focused on the similarities and differences of mental health reactions to COVID-19 between different countries. Therefore, the current study explores two general population samples collected in two different countries: Switzerland and Israel. These two countries are of particular interest as they entail several differences as well as similarities. Although the population size in these countries is very similar (8.57 million in Switzerland and 9.23 million in Israel), the sociopolitical climate, economic status, as well as mentality, are significantly different. Concerning the COVID-19 outbreak, both countries experienced significant health risks to the population, however, these challenges were dealt with differently by the two governments.

The aim of this study was to assess the association between COVID-19 related fears and negative affect as well as potential differences between the two countries. A moderated competitive mediation model was suggested, wherein country affiliation would moderate the direct and indirect paths. We hypothesized that a lower locus of control, higher fatalism, and higher perceived institutional betrayal would be associated with more COVID-19 related fear and more negative affect. We also assumed that the three types of control perceptions would mediate the association of COVID-19 related fear and negative affect. We also aimed to identify the most relevant control-related mediator in regards to negative emotions to determine potential starting points for interventions.

## Methods

### Participants and Procedure

This study was conducted during the peak of the COVID-19 outbreak in Switzerland and Israel when both countries were in lock-down. The educational systems were closed, classes took place online, and most people were working from home. In Israel, data collection took place from March 30 to May 16, 2020. During the initial stage of the data collection, there were 4,695 verified cases of COVID-19 and 16 deaths in Israel. By the end of the data collection, there were 16,607 verified cases and 268 deaths. During the majority of this time, the Israeli government had imposed quarantine on the entire population, apart from limited activities, such as healthcare and essential grocery shopping. In Israel, recent studies identified elevated levels of depression which were predicted by loneliness due to the social-distancing policy (37). In particular, COVID-19 related worries were associated with heightened anxiety and depression (38). Notably, during the COVID-19 outbreak, unemployment rates in Israel increased from 4% to ~27% of the population [1.276 million people; (39)].

In Switzerland, data collection commenced on April 24, when there were 29,014 verified cases of COVID-19 and 1,496 deaths. By the end of data collection on May 23, there were 30,628 verified COVID-19 cases and 1,677 deaths. While the population had not been required to be in quarantine, it was strongly recommended for people to remain at home during the time of the data collection. As in Israel, first studies conducted in Switzerland among student populations suggest that COVID-19 specific worries, lack of interaction and emotional support, and physical isolation were associated with negative mental health trajectories [e.g., (40)]. Unemployment rates in Switzerland were reported to be 3.3% in April and 3.4% in May. However, in April 2020, around one quarter of the working population had reduced working hours as a result of the government's action plan to control the negative impact of COVID-19 on the population (41).

A convenience sample of 595 Swiss and 639 Israeli participants was recruited via avenues of social media (e.g., Facebook) and a snowball technique. Participants were invited to participate in a study aiming to uncover psychosocial coping with challenges regarding COVID-19. Questionnaires were distributed electronically in local languages (i.e., German in Switzerland, and Hebrew and Arabic in Israel), using Unipark and Qualtrics Research Software. Inclusion criteria were a) above the age of 18, and b) fluent in the local language(s). The study was approved by the Institutional Review Boards in each country and all participants signed a consent form.

### Measures

Exposure to COVID-19 was assessed using 7 questions specifically tailored to assess COVID-19-related stressors (42). Participants were asked whether or not they were exposed to various COVID-19-related incidents (e.g., getting infected, quarantined, a family member got infected or quarantined, knowing someone who died from COVID-19). Overall exposure was calculated by summing all of the positive answers to exposure questions, with higher scores indicating higher exposure to COVID-19.

Fear of COVID-19 was evaluated by three questions specifically tailored to the COVID-19 experience (42). Participants were asked to rate the extent to which they fear the situations presented to them (“I am worried that I or my family could get infected or quarantined,” “I am afraid that the epidemic will spread widely and last long,” “I am afraid of the negative impact the COVID-19 will have on my life.”) on a five-point Likert scale, ranging between 1 (not at all) to 5 (very much). The fear of COVID-19 score was calculated by the summation of all of the responses to all items, with higher scores indicating higher COVID-19 fear. Cronbach's alphas were 0.71 and 0.76 for the Swiss and the Israeli samples, respectively, indicating acceptable reliability.

Fatalism was evaluated using the six-item Fatalism scale (43, 44). This scale assesses the degree to which one believes that destiny is externally determined, including two subscores: pessimistic and non-judgmental fatalism (44). Participants are asked to rate the extent to which each of the items is true for them, on a five-point Likert scale, ranging from 1 (strongly disagree) to 5 (strongly agree). Example items include: “I have learned that what is going to happen will happen,” “If bad things happen, it is because they were meant to happen.” The fatalism score was calculated by summing the responses to the six items, with higher scores indicating higher fatalism. Recent findings have demonstrated the scale's cross-cultural validity and reliability (44). In the current sample, Cronbach's alpha for the Swiss sample was 0.86 and 0.85 for the Israeli sample, indicating high reliability.

Health-related internal locus of control was measured with the Internal Health Locus of Control Scale [IHLC; (45)]. This six-item scale assesses the extent to which participants believe that their health is under their own control, determined by their own behavior. Participants were asked to rate, on a six-point Likert scale, the degree to which they agree with each item, ranging from 1 (strongly disagree) to 6 (strongly agree). Example items include: “I am in control of my health,” “If I take the right actions, I can stay healthy.” The locus of control score was calculated by summing the responses to all of the items, with higher scores indicating higher believed self-control. One item was omitted from the analysis due to a technical error. Nevertheless, this error did not appear to affect the reliability of the scale, as Cronbach's alpha for the Swiss sample was 0.84 and 0.87 for the Israeli sample, indicating high reliability.

Perceived institutional betrayal was assessed by a new questionnaire, partially based on the Institutional Betrayal Questionnaire—Health [IBQ-H; (46)]. The new questionnaire was adapted to measure the level to which participants perceived the local government and healthcare institutions as taking sufficient action in the face of the pandemic or, rather, betrayed their obligation to protect personal and public health and well-being. Respondents were instructed to report their agreement with each of the 12 items on a five-point Likert scale, ranging between 1 (completely disagree) to 5 (completely agree). For example: “The institutions betrayed your trust in them,” “Increased your risk of becoming sick/getting infected,” “Their actions reflect interests other than enhancing and protecting your health.” The total institutional betrayal score was calculated by the summation of the responses to all of the items. Cronbach's alphas for both the Swiss and Israeli samples were 0.91, indicating high reliability.

Negative affect was assessed using the negative affect subscale from the Positive and Negative Affect Schedule—Short form (PANAS; 48). The PANAS negative affect subscale consists of five emotions, including afraid, upset, and distressed. Participants were instructed to rate the extent to which they experienced each of the emotions during the last 2 weeks on a five-point Likert scale ranging between 1 (very little) to 5 (very much). The PANAS-negative affect score was calculated by summing the responses to all of the items, with higher scores indicating higher negative affect. Previous findings have documented the scale's validity and reliability (47). Cronbach's alphas were 0.81 and 0.89 for the Swiss and the Israeli samples, respectively, indicating high reliability.

### Data Analysis

First, the groups' background and demographic variables were compared. Additionally, independent sample *t*-tests were performed to assess the differences between the Swiss and Israeli samples in the main study variables. Next, Pearson correlation analyses were performed for each sample separately to assess the correlations between the study variables. Finally, we conducted a Multi-Group Path analysis in AMOS 23 software, which estimated the relation between fear of COVID-19 and negative affect as well as the indirect effects via fatalism, locus of control, and institutional betrayal. We also examined the model separately for the Israeli and Swiss samples. We controlled for age and gender and their associations with negative affect and the three mediators. We used age and gender because of differences between countries in these variables and since they were significantly associated with negative affect in both countries. The number of individuals in the household and education did not correlate with negative affect in either of the samples. The following indices were employed to determine whether the hypothesized models fit the data. A good model fit is indicated by a non-significant χ2, goodness-of-fit values as the comparative fit index (CFI), non-normed-fit index (NNFI), Tucker-Lewis Index (TLI) greater than .90, and a root-mean-square error of approximation (RMSEA) below 0.06 (48). We limited the paths between the countries to be significant and examined the differences between the χ^2^ of the constrained and free models. A significant χ^2Δ^ indicated that the paths were significant between the countries.

## Results

### Differences Between the Swiss Sample and the Israeli Sample

Background variables of the Swiss and Israeli samples are depicted in Table 1. As can be seen, some differences were found between the samples in terms of age, gender, education level, and the number of people in a household. Additionally, the Israeli sample reported an experience of significantly greater financial loss due to the COVID-19 outbreak, compared to the Swiss sample.

**Table 1:** Demographic characteristics by study group.

	**Swiss sample (*n* = 582)**	**Israeli sample (*n* = 639)**	**Chi square/ Independent sample *t*-test**
**Gender**			
Female	439 (73.8%)	534 (84.1%)	Chi square (1) = 19.77
Male	156 (26.2%)	101 (15.9%)	*p* < 0.001
Age^a^ (M, SD)	43.15, 14.77	47.25, 14.38	*t* (1,200) = 4.88 *p* < 0.001
**Education level**			
Primary/middle school	157 (26.4%)	3 (0.5%)	Chi square (2) = 235.54
Highschool	98 (16.5%)	42 (6.6%)	*p* < 0.001
Academic	340 (57.1%)	587 (92.9%)	
Number of people in household (M, SD)	2.68, 1.57	3.02, 1.58	*t* (1,211.44) = 3.81 *p* < 0.001
**Financial loss since COVID-19 outbreak**			
No financial loss	418 (70.5%)	123 (19.2%)	Chi square (2) = 331.08
Minor financial loss	135 (22.8%)	356 (55.7%)	*p* < 0.001
Major financial loss	40 (6.7%)	160 (25%)	

a *Age range: 18–99 years. Age distribution: 18–29 years (Switzerland: n = 132, 22.2%; Israel n = 62, 9.7%) 30–59 years (Switzerland n = 357, 60.0%); Israel n = 413, 64.6%), 60–99 (Switzerland: n = 104, 15.5%; Israel n = 134, 21%)*.

As can be seen in Table 2, although no differences were found between the Swiss and Israeli samples in exposure to COVID-19 related stressors, significant differences were found between the two samples in all of the study variables. Specifically, the findings revealed that, compared to the Swiss sample, the Israeli sample experienced a higher fear of COVID-19 as well as higher fatalism, locus of control, and negative affect. Notably, the Israeli sample reported remarkably higher levels of perceived institutional betrayal than the Swiss sample.

**Table 2:** Study variables by study group.

	**Swiss sample (*n* = 595)**	**Israeli sample (*n* = 639)**	**Independent sample *t*-test**
Exposure to COVID-19 (M, SD)	1.41, 1.44	1.38, 1.15	*t*-test (1,204) = 0.3 *p* = 0.77
Fear of COVID-19 (M, SD)	6.32, 1.82	7.82, 2.01	*t*-test (1,232) = 13.68 *p* < 0.001
Fatalism (M, SD)	15.02, 5.65	16.1, 5.11	*t*-test (1,232) = 3.53 *p* < 0.0015
Locus of control (M, SD)	20.38, 4.32	22.72, 4.43	*t*-test (1,229) = 9.45 *p* < 0.001
Institutional betrayal (M, SD)	19.13, 8.79	34.68, 9.33	*t*-test (1,232) = 30.1 *p* < 0.001
Negative affect (M, SD)	10.09, 4.01	12.24, 4.84	*t*-test (1,232) = 8.47 *p* < 0.001

*Exposure to COVID-19 range: 0–7; Fear of COVID-19: 3–12; Fatalism range: 0–30; Locus of control range: 5–35; Institutional betrayal range: 12–55; Negative affect: 4.86–25*.

### Intercorrelations Between the Study Variables

As depicted in Table 3, the analyses revealed that exposure to COVID-19 was correlated with fear of COVID-19 in the Swiss sample, however, this was not found in the Israeli sample. Among both the Swiss and Israeli samples, fear of COVID-19 was inversely correlated with locus of control and positively correlated with institutional betrayal and negative affect. A significant inverse correlation between fear of COVID-19 and fatalism was observed only in the Swiss sample. Finally, in both samples, negative affect was inversely correlated with locus of control and positively correlated with institutional betrayal.

**Table 3:** Intercorrelations between study variables.

	**Exposure to COVID-19**	**Fear of COVID-19**	**Fatalism**	**Locus of control**	**Institutional betrayal**	**Negative affect**
Exposure to COVID-19	1	0.13^**^	−0.074	−0.06	0.004	0.074
Fear of COVID-19	0.004	1	−0.12^**^	−0.26^***^	0.09^*^	0.52^***^
Fatalism	−0.084^*^	0.009	1	0.19^***^	0.15^***^	0.024
Locus of control	−0.025	−0.12^**^	0.14^***^	1	0.14^**^	−0.12^**^
Institutional betrayal	−0.053	0.094^*^	−0.024	0.07	1	0.32^***^
Negative affect	0.01	0.54^***^	0.045	−0.16^***^	0.13^**^	1

*Results above diagonal reflect intercorrelations among Swiss sample, and results under diagonal reflect intercorrelations among Israeli sample*.

* p < 0.05;

** p < 0.01;

*** *p < 0.001*.

### Moderated Mediation

We assessed whether the association between fear of COVID-19 and negative affect differed between the Israeli and Swiss samples. Additionally, we examined the potential mediating role of fatalism, locus of control, and perceived institutional betrayal. We controlled for the effects that age and gender bear for negative affect and for the three mediators, fatalism, locus of control, and perceived institutional betrayal. To this end, we ran multigroup path analysis models that estimated the relation between fear of COVID-19 and negative affect, and the indirect effects via fatalism, locus of control, and institutional betrayal, controlling for age and gender, separately for the Israeli and Swiss samples. The multigroup model fit the overall data well, χ^2^(*N* = 1,234, *df* = 16) = 76.61, *p* < 0.001, CFI = 0.91, NNFI = 0.93, TLI = 0.92, RMSEA = 0.055, 90% CI [0.042, 0.068]. However, the model fit the data only adequately for each individual sample though in both samples RMSEA was high: χ^2^(*N* = 639, *df* = 8) = 33.72, *p* < 0.001, CFI = 0.91, NNFI = 0.93, TLI = 0.93, RMSEA = 0.071, 90% CI [0.047, 0.097] for the Israeli sample and, χ^2^(*N* = 595, *df* = 8) = 42.89, *p* < 0.001, CFI = 0.91, NNFI = 0.93, TLI = 0.92, RMSEA = 0.086, 90% CI [0.062, 0.112] for the Swiss sample (see Supplementary Materials 1). In both samples higher age was associated with lower negative affect and being male was related to lower levels of negative affect compared to being female. In both samples, being male was associated with lower fatalism, but gender was not related to locus of control of institutional betrayal. In the Israeli sample, higher age was related to higher institutional betrayal and lower fatalism, but it was not related to locus of control. However, in the Swiss sample, higher age was associated with higher fatalism, but not with institutional betrayal or locus of control. Since the model fit was not optimal, we then examined a nested model.

**Figure 1:**
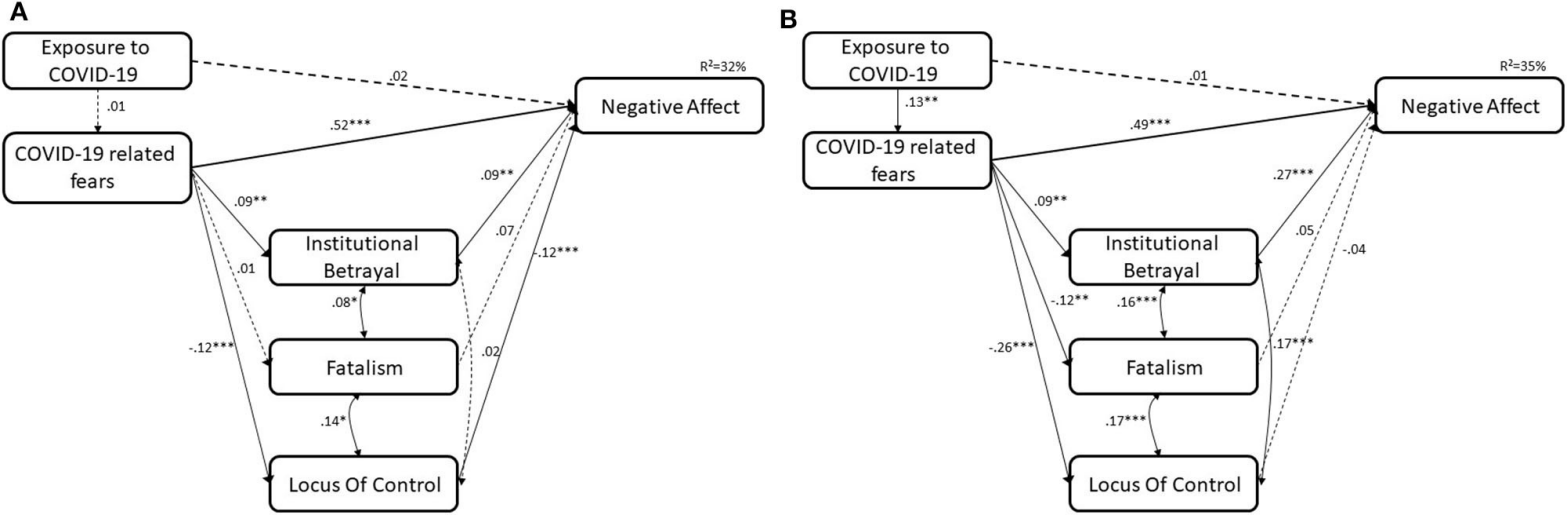
**(A)** Israeli sample. **(B)** Swiss sample. Full lines represent significant paths. Dashed lines represent insignificant paths ***p < 0.001; **p < 0.01; *p < 0.05.

The control variables were removed from the models and excellent model fit was found with similar effects, both in estimates' direction and intensity. The multigroup model fit the overall data well, χ^2^(*N* = 1234, *df* = 6) = 8.87, *p* = 0.018, CFI = 0.99, NNFI = 0.99, TLI = 1.00, RMSEA =.02, 90% CI [0.000, 0.045], as well as data collected from each sample: χ^2^(*N* = 639, *df* = 3) = 6.59, *p* = 0.090, CFI = 0.99, NNFI = 0.98, TLI = 0.99, RMSEA = 0.043, 90% CI [0.000, 0.089] for the Israeli sample and, χ^2^(*N* = 595, *df* = 3) = 2.28, *p* = 0.520, CFI = 1.00, NNFI = 1.00, TLI = 1.01, RMSEA = 0.000, 90% CI [0.000, 0.062] for the Swiss sample. The differences between the models' Chi square was not significant, (p's ranged 0.23 to 0.69), which indicates that the more parsimonious model is favorable (Figure 1).

The analysis revealed that for both the Israeli and Swiss samples, higher levels of fear of COVID-19 were related to higher levels of negative affect. This path was not significant between the groups, Δχ^2^(7) = 10.79, *p* = 0.150. In both the Israeli and Swiss samples, higher fear of COVID-19 was related to higher institutional betrayal. This path was equal between the samples, Δχ^2^(7) = 8.87, *p* = 0.262. However, the samples differed in regard to the associations between fear of COVID-19 and fatalism and locus of control. In both the Israeli and Swiss samples, the path between fear of COVID-19 and locus of control was significant, indicating that higher fear of COVID-19 was related to lower locus of control, although in the Swiss sample it was stronger, Δχ^2^(7) = 17.11, *p* = 0.017. A difference between the samples was found in the associations between fear of COVID-19 and fatalism. While in the Israeli sample this path was not significant, in the Swiss sample it was significant and showed that higher fear of COVID-19 was related to lower fatalism, Δχ^2^(7) = 15.08, *p* = 0.035.

The relationship between institutional betrayal and negative affect was significant in both samples, indicating that higher institutional betrayal was associated with higher negative affect, although this path was significantly stronger in the Swiss sample, Δχ^2^(7) = 18.96, *p* = 0.008. In the Swiss sample, the associations between fatalism and lower locus of control, on the one hand, and negative affect, on the other hand, were insignificant. However, in the Israeli sample, the path between lower locus of control and negative affect was significant, indicating that a higher locus of control was associated with lower negative affect. The difference between samples in this path was not significant, Δχ^2^(7) = 12.33, *p* = 0.090. In addition, the association between fatalism and negative affect was marginally significant in the Israeli sample. The difference between the samples in this path was not significant, Δχ^2^(7) = 9.11, *p* = 0.245.

The total indirect effect (comprised of the sum of the three indirect effects) was significant in the Israeli sample (total indirect effect: Estimate =.06, *se* = 0.02, 95% CI [0.0270, 0.0980]) but insignificant in the Swiss sample (total indirect effect: Estimate =.06, *se* = 0.04, 95% CI [−0.0070, 0.1190]). The indirect effects via fatalism were not significant in either sample (all 95% CI included 0). However, the indirect effects via institutional betrayal were significant in both the Israeli (indirect effect: Estimate = 0.02, *se* = 0.01, 95% CI [0.0020, 0.0460]) and the Swiss (indirect effect: Estimate = 0.05, *se* = 0.03, 95% CI [0.0140, 0.1150]) samples. The indirect effect via locus of control (indirect effect: Estimate = 0.03, *se* = 0.01, 95% CI [0.0080, 0.0630]) was significant in the Israeli sample but not in the Swiss sample (indirect effect: Estimate = −0.01, *se* = 0.02, 95% CI [−0.0560, 0.0260]).

The results indicate that there are moderated mediations with the country as the moderator. In the Israeli and Swiss samples, higher levels of fear of COVID-19 were related to higher institutional betrayal, which was associated with a higher negative affect. In the Israeli sample, higher levels of fear of COVID-19 were related to higher locus of control, which was associated with higher negative affect. Fatalism did not mediate the path between fear of COVID-19 and negative affect.

## Discussion

It is a basic human instinct to strive for control when adversity strikes. In this study, we sought to explore three different control perceptions (fatalism, internal locus of control, and perceived institutional betrayal) as potential mediators of the association between COVID-19 related fear and negative affect in two samples collected during the lockdown periods in Israel and Switzerland. This study aims to contribute to our understanding of the mechanisms associated with negative affect in the general population during a global health crisis and to better understand the role the local context plays in the stress response. The results revealed that perceived institutional betrayal was the strongest mediator of the association between COVID-19 related fear and negative affect, which was significant in both samples. In addition, health related internal locus of control was a mediator among the Israeli sample only.

As was found in previous studies [e.g., (2, 5)], the association between COVID-19 related fears and negative affect was substantial in both samples, corresponding to a medium-large effect size. In contrast, actual exposure to COVID-19 was unrelated to negative emotions among individuals from both countries, which suggests that in the context of COVID-19, subjective appraisals rather than objective threats determined emotional adjustment. Similar observations have previously been made across a variety of contexts related to impaired physical health, such as among cancer patients [e.g., (49)]. Although self-rated exposure to COVID-19 was equal in the two samples, distress levels were different. Israeli participants reported significantly higher COVID-19 related fear and more negative affect.

### The Role of Institutional Betrayal

Perceived institutional betrayal was the concept of interest that explained most of the variance in the current model. In both Switzerland and Israel, higher COVID-19 related fears were associated with reduced trust in local government and healthcare institutions to protect against the virus and a higher perceived institutional betrayal was associated with more negative emotions. In addition, institutional betrayal mediated the association between COVID-19 related fears and negative affect in both samples. These findings highlight the central role of the authorities in an individual's mental well-being during times of crisis. In a situation as threatening as a pandemic, people turn toward the authorities whose responsibility includes supporting and protecting the individual. If such support is not granted, it is a grave source of distress. The current results thus suggest that in order to mitigate the negative psychosocial consequences of COVID-19, special attention should be paid to strengthening trust in the authorities as this has the potential to buffer the negative impact of fears. Future research should formally explore the specific factors that influence perceptions of institutional betrayal and develop appropriate intervention strategies. The ongoing pandemic offers the chance to learn important lessons that may serve to improve general crisis management in the future.

Interestingly, there was a striking difference in perceived institutional betrayal in the two samples with significantly higher mean values in Israel (*M* = 34.7) compared to Switzerland (*M* = 19.2). It is likely that an important contributor to the conspicuously high levels of institutional betrayal in Israel was the economic difficulties the country encountered during the lockdown period. While in Switzerland unemployment rates remained stable during the data collection phase, in Israel they increased from 4% to ~27% and, consequently, Israelis reported higher financial loss compared to the Swiss participants (see Table 1). In support of this explanation, recent findings have shown that Israelis who received more financial compensation from the government during the lockdown were more likely to comply with the imposed restrictions (50). Additionally, the significantly higher perceived institutional betrayal among Israelis may also reflect circumstances predating the COVID-19 crisis, such as political turmoil and related distrust in the political leadership, which may also have decreased Israelis trust in government and healthcare institutions. Switzerland, on the other hand, did not experience political unrest before the pandemic. The high levels of perception of institutional betrayal could at least partially explain why the Israeli sample suffered from higher fear and negative emotions during the study period, despite lower numbers of infections and deaths due to COVID-19.

### The Role Internal Locus of Control

In line with the hypotheses, less fear of COVID-19 was associated with higher health locus of control in both samples, thereby extending findings by Brailovskaia and Margraf (30) who showed negative associations of general (not health-related) sense of control and burden by COVID-19. However, even though health locus of control was correlated with negative affect, in Switzerland it did not mediate the association of interest in the path model. This finding suggests that among the Swiss, health locus of control did not explain variance in negative affect above and beyond the other study variables. In fact, the only control perception that was associated with negative affect in the Swiss model was institutional betrayal. Contrarily, in Israel, health locus of control also mediated the association of fear and negative affect. It may be speculated that surviving multiple wars and adversities may have enabled a “survivor” identity, in which it is particularly important to take personal, active control in the face of these difficulties (51, 52). As such, it is possible that the sense of personal control over one's health is particularly relevant for Israelis when facing an uncontrollable stressor, such as the COVID-19 pandemic. As a second explanation, it may be speculated that in the face of high perceived institutional betrayal personal means of control may become more important. The significant and positive correlation of perceived institutional betrayal and locus of control indicates that this may be the case.

### The Role of Fatalism

In Switzerland, COVID-19 related fear was associated with increased fatalism. This is in line with previous research that found negative associations of fatalism and anxiety [e.g., (22, 53)]. From a self-regulation perspective, disengaging from perceptions of control may resolve the conflict that arises from the insecurities related to a new situation, such as the COVID-19 crisis, in which an individual has little control over the course of events (22). Hayes and Clerk (54) conducted an experimental study which showed that COVID-19 related fatalism could be deliberately influenced by manipulating control beliefs. While a fatalistic message arguing that the pandemic is unstoppable and that mitigation efforts may do more harm than good increased fatalism, an optimistic message that drew attention to the effectiveness of coping efforts and collective connectedness in times of need reduced fatalism. Furthermore, several recent studies reported that more fatalistic beliefs about the infectiousness of COVID-19 were less likely to comply with preventive measures (55, 56). In Israel, however, higher fear of COVID-19 was unrelated to fatalism, which differs from the findings in the Swiss sample as well as the US sample of Hayes and Clerk (54). One explanation for these differences could be embedded in the cultural differences between the two countries. As described above, the geopolitical circumstances in Israel are complex and since its establishment, the Palestinian and Israeli populations have faced ongoing tension and conflict. This prolonged sense of threat may have resulted in higher general fatalism, which was unaffected by COVID-19 related fears. Indeed, the Israeli sample reported significantly higher fatalism than the Swiss sample. Previous theorists have described fatalism as a social axiom (57), which suggests that it develops through the interaction of a cognitively and emotionally active person and his or her socially structured environment (58). As such, cultural differences regarding the function of fatalism seem to be explainable. Indeed, previous research has shown significant mean-level differences in fatalism between different European and African countries (44).

Finally, contrary to our hypothesis, higher fatalism was not associated with a stronger negative affect in either of the samples and also did not represent a mediator in the current model. Despite previous findings, which have shown strong positive associations between fatalism with psychological distress (24) and depression (54), in the current study no such effect was found. Although in the context of adversity fatalism is generally considered a risk factor for mental health and well-being, the data suggest that during the COVID-19 pandemic this was not the case. Further research is necessary to uncover the association between fatalism and distress, including an exploration of its underlying explanatory mechanisms.

Several limitations should be acknowledged when interpreting the current findings. First, the samples were recruited via social media and, therefore, are not representative of the Swiss and Israeli populations, which limits the generalization of the results. Additionally, females were overrepresented in the sample and participants were relatively highly educated. Second, the cross-sectional nature of the data does not allow for any inferences on causality. Third, the study relied on self-report data rather than clinician-administered interviews. Due to the urgency of the COVID-19 pandemic, the questionnaires assessing institutional betrayal as well as COVID-19 exposure and fear had not been validated in Israeli and Swiss populations. Finally, comorbid mental health problems are likely related to negative affect during the COVID-19 pandemic but have not been considered in the current models. Nevertheless, given the timeliness of the research question and the urgency of understanding negative emotional reactions in the context of the COVID-19 pandemic, this first examination yielded important exploratory information on predictors of COVID-19 related mental health burdens. Future research should evaluate how they relate to other risk factors, such as temperament traits and related personality constructs, which have been shown to be relevant to the mental health response to COVID-19 (59).

Within the framework of these empirical findings, it can be concluded that the reaction of the authorities appears to be of crucial importance with regard to the emotional state and well-being of the population in both countries. As international experts warn of a possible rise in mental health problems in the aftermath of COVID-19 (11, 60, 61), a vital next step would be to closely investigate the factors accounting for the perceptions of institutional betrayal in order to take measures to lower it and, thereby, also buffer the negative impact of the COVID-19 crisis on people's mental health. However, the findings emphasize that, even though COVID-19 was associated with fear as well as negative affect in Israel and Switzerland, significant differences were also identified. The current results thus suggest that, in Israel, interventions strengthening the health locus of control would have more potential as a means to stop the spill-over from specific fears to negative affect. Presuming replications of these findings, strengthening the health locus of control would be a potential intervention target. Despite the fact that COVID-19 is a global phenomenon, prevention and intervention strategies should be adjusted to local contexts.

## Data Availability Statement

The raw data supporting the conclusions of this article will be made available by the authors, without undue reservation.

## Ethics Statement

The studies involving human participants were reviewed and approved by Institutional Review Boards of the University of Zurich and Tel Aviv University. The patients/participants provided their written informed consent to participate in this study.

## Author Contributions

RB: contributed to the conceptualization and design of the study, collected the data in Switzerland, and drafted the manuscript. NT: contributed to the conceptualization and the design of the study, collected the data in Israel, contributed to the data analysis and reporting, and conducted a critical review of the manuscript. YL: contributed to the conceptualization and the design of the study, contributed to the data analysis and reporting, and conducted a critical review of the manuscript. HA-R and AM: contributed to the conceptualization and the design of the study and conducted a critical review of the manuscript. All authors contributed to the article and approved the submitted version.

## Conflict of Interest

The authors declare that the research was conducted in the absence of any commercial or financial relationships that could be construed as a potential conflict of interest.

## Footnotes

^1^https://www.researchgate.net/publication/340763183_Anxiety_Depression_Traumatic_Stress_and_COVID-19_Related_Anxiety_in_the_UK_General_ Population_During_the_COVID-19_Pandemic (accessed October 13, 2020).

## References

[1] World Health Organization (2020) Coronavirus Disease 2019 (COVID-19): Situation Report 85. Available online at: https://www.who.int/emergencies/diseases/novel-coronavirus-2019/situation-reports (accessed May 11, 2020).

[2] Ahorsu DK Lin CY Imani V Saffari M Griffiths MD Pakpour AH. The fear of COVID-19 scale: development and initial validation. Int J Ment Health Addict. (2020) 1–9. 10.1007/s11469-020-00270-8. [Epub ahead of print]. 32226353 PMC7100496

[3] Bareket-Bojmel L Shahar G Margalit M. COVID-19-related economic anxiety is as high as health anxiety: findings from the USA, the UK, and Israel. Int J Cogn Ther. (2020) 10:1–9. 10.1007/s41811-020-00078-3 32837674 PMC7258609

[4] Lee SA Mathis AA Jobe MC Pappalardo EA. Clinically significant fear and anxiety of COVID-19: a psychometric examination of the coronavirus anxiety scale. Psychiatry Res. (2020) 290:113112. 10.1016/j.psychres.2020.113112 32460185 PMC7237368

[5] Pakpour AH Griffiths MD The fear of COVID-19 and its role in preventive behaviors. J. Concurr. Disord. (2020) 2:58–63. Available online at: https://concurrentdisorders.ca/2020/04/03/the-fear-of-covid-19-and-its-role-in-preventive-behaviors/ (accessed June 9, 2020).

[6] Lai J Ma S Wang Y Cai Z Hu J Wei N. Factors associated with mental health outcomes among health care workers exposed to coronavirus disease 2019. JAMA Netw open. (2020) 3:e203976. 10.1001/jamanetworkopen.2020.3976 32202646 PMC7090843

[7] Casagrande M Favieri F Tambelli R Forte G. The enemy who sealed the world: Effects quarantine due to the COVID-19 on sleep quality, anxiety, and psychological distress in the Italian population. Sleep Med. (2020) 75:12–20. 10.1016/j.sleep.2020.05.011 32853913 PMC7215153

[8] Satici B Saricali M Satici SA Griffiths MD. Intolerance of uncertainty and mental wellbeing: serial mediation by rumination and fear of COVID-19. Int J Ment Health Addict. (2020). 10.1007/s11469-020-00305-0. [Epub ahead of print]. 32427165 PMC7228430

[9] Forte G Favieri F Tambelli R Casagrande M. COVID-19 pandemic in the italian population: Validation of a post-traumatic stress disorder questionnaire and prevalence of PTSD symptomatology. Int J Environ Res Public Health. (2020) 17:4151. 10.3390/ijerph17114151 32532077 PMC7312976

[10] Ornell F Schuch JB Sordi AO Kessler FHP. “Pandemic fear” and COVID-19: mental health burden and strategies. Braz J Psychiatry. (2020) 42:232–5. 10.1590/1516-4446-2020-0008 32267343 PMC7236170

[11] Sani G Janiri D Di Nicola M Janiri L Ferretti S Chieffo D. Mental health during and after the COVID-19 emergency in Italy. Psychiatry Clin Neurosci. (2020) 74:372–372. 10.1111/pcn.13004 32248608

[12] Shigemura J Ursano RJ Morganstein JC Kurosawa M Benedek DM. Public responses to the novel 2019 coronavirus (2019-nCoV) in Japan: Mental health consequences and target populations. Psychiatry Clin Neurosci. (2020) 74:281–2. 10.1111/pcn.12988 32034840 PMC7168047

[13] Wang C Pan R Wan X Tan Y Xu L Ho CS. Immediate psychological responses and associated factors during the initial stage of the 2019 coronavirus disease (COVID-19) epidemic among the general population in China. Int J Environ Res Public Health. (2020) 17:1729. 10.3390/ijerph17051729 32155789 PMC7084952

[14] Forte G Favieri F Tambelli R Casagrande M. The enemy which sealed the world: Effects of COVID-19 diffusion on the psychological state of the Italian population. Cournal Clin Med. (2020) 9:1802. 10.3390/jcm9061802 32531884 PMC7356935

[15] Maunder R Hunter J Vincent L Bennett J Peladeau N Leszcz M. The immediate psychological and occupational impact of the 2003 SARS outbreak in a teaching hospital. CMAJ. (2003) 168:1245–51. 12743065 PMC154178

[16] Cao W Fang Z Hou G Han M Xu X Dong J. The psychological impact of the COVID-19 epidemic on college students in China. Psychiatry Res. (2020) 287:112934. 10.1016/j.psychres.2020.112934 32229390 PMC7102633

[17] González-Sanguino C Ausín B Castellanos MÁ Saiz J López-Gómez A Ugidos C. Mental health consequences during the initial stage of the 2020 coronavirus pandemic (COVID-19) in Spain. Brain Behav Immun. (2020) 87:172–6. 10.1016/j.bbi.2020.05.040 32405150 PMC7219372

[18] Mowbray H. In beijing, coronavirus 2019-nCoV has created a siege mentality. Br J Med. (2020) 368:m516. 10.1136/bmj.m516 32033967

[19] Torales J O'Higgins M Castaldelli-Maia JM Ventriglio A. The outbreak of COVID-19 coronavirus and its impact on global mental health. Int J Soc Psychiatry. (2020) 66:317–20. 10.1177/0020764020915212 32233719

[20] Ryan RM Deci EL. Self-Determination Theory: Basic Psychological Needs in Motivation, Development, and Wellness. New York, NY (2017).

[21] Solomon RC On fate and fatalism. Philos East West. (2003) 53:435–54. 10.1353/pew.2003.0047

[22] Hayes J Ward CLP McGregor I. Why bother? Death, failure, and fatalistic withdrawal from life. J Pers Soc Psychol. (2016) 110:96–115. 10.1037/pspp0000039 25915129

[23] Roberts RE Roberts CR Chen IG. Fatalism and risk of adolescent depression. Psychiatry. (2000) 63:239–52. 10.1080/00332747.2000.11024917 11125670

[24] Ross CE Mirowsky J Cockerham WC. Social class, mexican culture, and fatalism: their effects on psychological distress. Am J Community Psychol. (1983) 11:383–99. 10.1007/BF00894055 6637901

[25] Levenson H (1981). Differentiating among internality, powerful others, and chance. In: Lefcourt HM, editor. Research with the Locus of Control Construct (New York, NY: Academic). p. 15–63.

[26] Groth N Schnyder N Kaess M Markovic A Rietschel L Moser S. Coping as a mediator between locus of control, competence beliefs, and mental health: a systematic review and structural equation modelling meta-analysis. Behav Res Ther. (2019) 121:103442. 10.1016/j.brat.2019.103442 31430689

[27] Bandura A Schunk DH Cultivating competence, self-efficacy, and intrinsic interest through proximal self-motivation. J Pers Soc Psychol. (1981) 41:586–98. 10.1037/0022-3514.41.3.586

[28] Cheng C Cheung MWL Lo BCY. Relationship of health locus of control with specific health behaviours and global health appraisal: a meta-analysis and effects of moderators. Health Psychol Rev. (2016) 10:460–77. 10.1080/17437199.2016.1219672 27556686 PMC5214986

[29] Yeoh SH Tam CL Wong CP Bonn G. Examining depressive symptoms and their predictors in Malaysia: stress, locus of control, and occupation. Front Psychol. (2017) 8:1411. 10.3389/fpsyg.2017.01411 28878710 PMC5572380

[30] Brailovskaia J Margraf J. Predicting adaptive and maladaptive responses to the Coronavirus (COVID-19) outbreak: a prospective longitudinal study. Int J Clin Heal Psychol. (2020) 20:183–91. 10.1016/j.ijchp.2020.06.002 32837518 PMC7321043

[31] Smith CP Freyd JJ. Institutional betrayal. Am Psychol. (2014) 69:575–84. 10.1037/a0037564 25197837

[32] Pinciotti CM Orcutt HK. Institutional betrayal: who is most vulnerable? J Interpers Violence. (2019) 10.1177/0886260518802850. [Epub ahead of print]. 30264672

[33] Smith CP Freyd JJ. Dangerous safe havens: institutional betrayal exacerbates sexual trauma. J Trauma Stress. (2013) 26:119–24. 10.1002/jts.21778 23417879

[34] Monteith LL Bahraini NH Matarazzo BB Soberay KA Smith CP. Perceptions of institutional betrayal predict suicidal self-directed violence among veterans exposed to military sexual trauma. J Clin Psychol. (2016) 72:743–55. 10.1002/jclp.22292 27007795

[35] Gold JA. Covid-19: adverse mental health outcomes for healthcare workers. BMJ. (2020) 369:m1815. 10.1136/bmj.m1815 32371465

[36] Klest B Smith CP May C McCall-Hosenfeld J Tamaian A. COVID-19 has united patients and providers against institutional betrayal in health care: a battle to be heard, believed, and protected. Psychol Trauma. (2020) 12:159–61. 10.1037/tra0000855 32478553

[37] Palgi Y Shrira A Ring L Bodner E Avidor S Bergman Y. The loneliness pandemic: loneliness and other concomitants of depression, anxiety and their comorbidity during the COVID-19 outbreak. J Affect Disord. (2020) 275:109–11. 10.1016/j.jad.2020.06.036 32658811 PMC7330569

[38] Barzilay R Moore TM Greenberg DM DiDomenico GE Brown LA White LK. Resilience, COVID-19-related stress, anxiety and depression during the pandemic in a large population enriched for healthcare providers. Transl Psychiatry. (2020) 10:291. 10.1038/s41398-020-00982-4 32820171 PMC7439246

[39] Centrel Bureau of Statistics I Employment Rates. (2020). Available online at: https://www.cbs.gov.il/EN/Pages/default.aspx (Accessed July 28, 2020).

[40] Elmer T Mepham K Stadtfeld C. Students under lockdown: comparisons of students' social networks and mental health before and during the COVID-19 crisis in Switzerland. PLoS ONE. (2020) 15:e0236337. 10.1371/journal.pone.0236337 32702065 PMC7377438

[41] Federal Statistical Office Unemployment Statistics. (2020). Available online at: https://www.arbeit.swiss/secoalv/de/home/menue/institutionen-medien/statistiken.html (Accessed July 27, 2020).

[42] Zhen R Zhou X Predictive factors of public anxiety under the outbreak of COVID-19. Chinese J Appl Psychol. (2020) 26:99–107

[43] Esparza OA Wiebe JS Quiñones J. Simultaneous development of a multidimensional fatalism measure in english and Spanish. Curr Psychol. (2015) 34:597–612. 10.1007/s12144-014-9272-z 26770053 PMC4703606

[44] Maercker A Ben-Ezra M Esparza OA Augsburger M. Fatalism as a traditional cultural belief potentially relevant to trauma sequelae: measurement equivalence, extent and associations in six countries. Eur J Psychotraumatol. (2019) 10:1657371. 10.1080/20008198.2019.1657371 31528270 PMC6735334

[45] Wallston KA Strudler Wallston B DeVellis R. Development of the multidimensional health locus of control (MHLC) scales. Health Educ Monogr. (1978) 6:160–70. 10.1177/109019817800600107 689890

[46] Smith CP. First, do no harm: institutional betrayal and trust in health care organizations. J Multidiscip Healthc. (2017) 10:133–44. 10.2147/JMDH.S125885 28435281 PMC5388348

[47] Mackinnon A Jorm AF Christensen H Korten AE Jacomb PA Rodgers B A short form of the positive and negative affect schedule: evaluation of factorial validity and invariance across demographic variables in a community sample. Pers Individ Dif. (1999) 27:405–16. 10.1016/S0191-8869(98)00251-7

[48] Hu LT Bentler PM Cutoff criteria for fit indexes in covariance structure analysis: conventional criteria versus new alternatives. Struct Equ Model. (1999) 6:1–55. 10.1080/10705519909540118

[49] Hamama-Raz Y Solomon Z Schachter J Azizi E. Objective and subjective stressors and the psychological adjustment of melanoma survivors. Psychooncology. (2007) 16:287–94. 10.1002/pon.1055 16858669

[50] Bodas M Peleg K. Self-isolation compliance in the COVID-19 era influenced by compensation: findings from a recent survey in Israel. Health Aff. (2020) 39:936–41. 10.1377/hlthaff.2020.00382 32271627

[51] Amara M Schnell I Identity repertoires among arabs in Israel. J Ethn Migr Stud. (2004) 30:175–93. 10.1080/1369183032000170222

[52] Weissbrod L Israeli Identity: In Search of a Successor to the Pioneer, Tsabar and Settler. New York, NY: Routledge/Taylor & Francis Group (2014).

[53] Shahid F Beshai S Del Rosario N. Fatalism and depressive symptoms: active and passive forms of fatalism differentially predict depression. J Relig Health. (2020). 10.1007/s10943-020-01024-5. [Epub ahead of print]. 32441015

[54] Hayes J Clerk L Fatalism in the Fight Against COVID-19: Implications for Mitigation and Mental Health (Working Paper). (2020). Available online at: https://www.researchgate.net/publication/340979910_Fatalism_in_the_Fight_against_COVID-19_Implications_for_Mitigation_and_Mental_Health (accessed September 15, 2020).

[55] Jimenez T Restar A Helm PJ Cross RI Barath D Arndt J. Fatalism in the context of COVID-19: perceiving coronavirus as a death sentence predicts reluctance to perform recommended preventive behaviors. SSM Popul Health. (2020) 11:100615. 10.1016/j.ssmph.2020.100615 32572381 PMC7278631

[56] Papageorge NW Zahn MV Jamison JC Tripodi E Zahn MV Jamison JC Socio-Demographic Factors Associated With Self-Protecting Behavior During the COVID-19 Pandemic. Cambridge: National Bureau of Economic Research (2020). 10.1007/s00148-020-00818-x PMC7807230 33462529

[57] Leung K Bond MH De Carrasquel SR Munoz C Hernández M Murakami F Social axioms: the search for universal dimensions of general beliefs about how the world functions. J Cross Cult Psychol. (2002) 33:286–302. 10.1177/0022022102033003005

[58] Hui C-M Hui H-HN The mileage from social axioms: Learning from the past and looking forward. In: Leung K, Bond MH, editors. Psychological Aspects of Social Axioms. New York, NY: Springer (2009). p. 13–30. 10.1007/978-0-387-09810-4_2

[59] Moccia L Janiri D Pepe M Dattoli L Molinaro M De Martin V. Affective temperament, attachment style, and the psychological impact of the COVID-19 outbreak: an early report on the Italian general population. Brain Behav Immun. (2020) 87:75–9. 10.1016/j.bbi.2020.04.048 32325098 PMC7169930

[60] da Silva Lopes BC Jaspal R. Understanding the mental health burden of COVID-19 in the United Kingdom. Psychol Trauma Theory, Res Pract Policy. (2020) 12:465–7. 10.1037/tra0000632 32478547

[61] Holmes EA O'Connor RC Perry VH Tracey I Wessely S Arseneault L. Multidisciplinary research priorities for the COVID-19 pandemic: a call for action for mental health science. Lancet Psychiatry. (2020) 7:547–60. 10.1016/S2215-0366(20)30168-1 32304649 PMC7159850

