# Supplementary figures and images for: Negative Affect, Fatalism, and Perceived Institutional Betrayal in Times of the Coronavirus Pandemic: A Cross-Cultural Investigation of Control Beliefs
Source: Front Psychiatry. 2020 Oct 26;11:589914. doi: 10.3389/fpsyt.2020.589914 (PMC7649425; doi:10.3389/fpsyt.2020.589914)

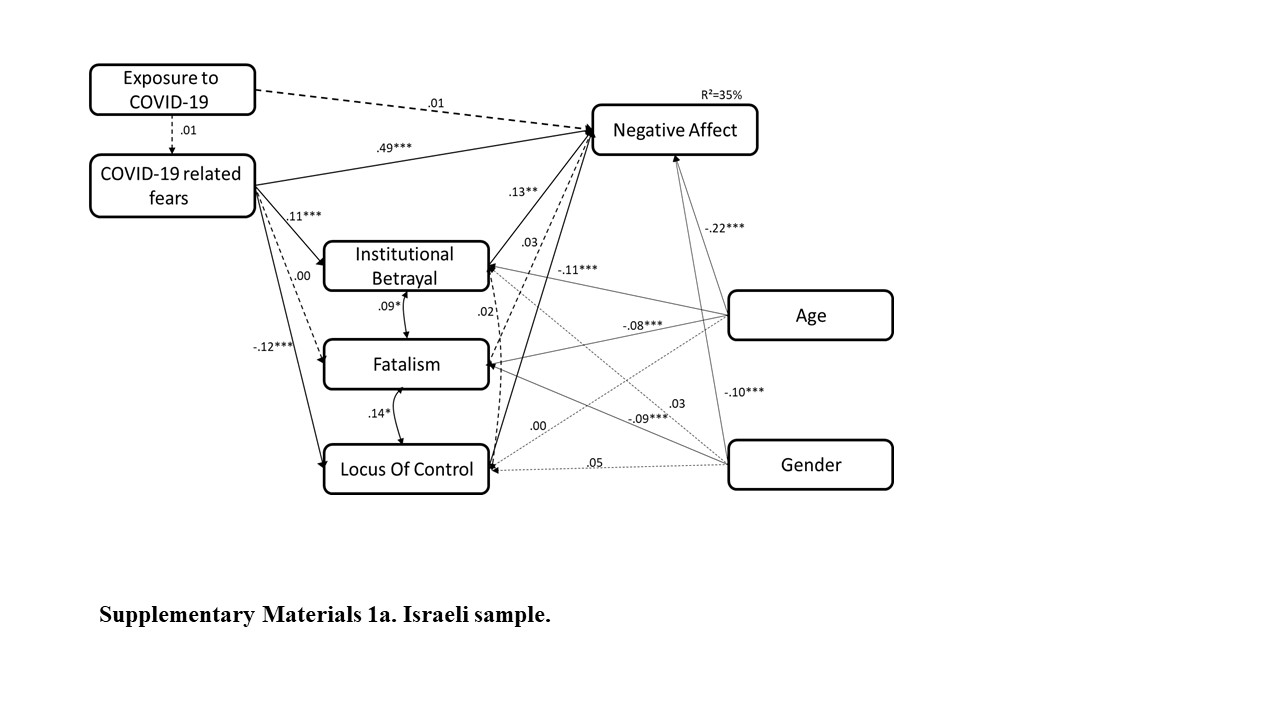

Supplement: Supplementary file 1 [file Image_1.JPEG]

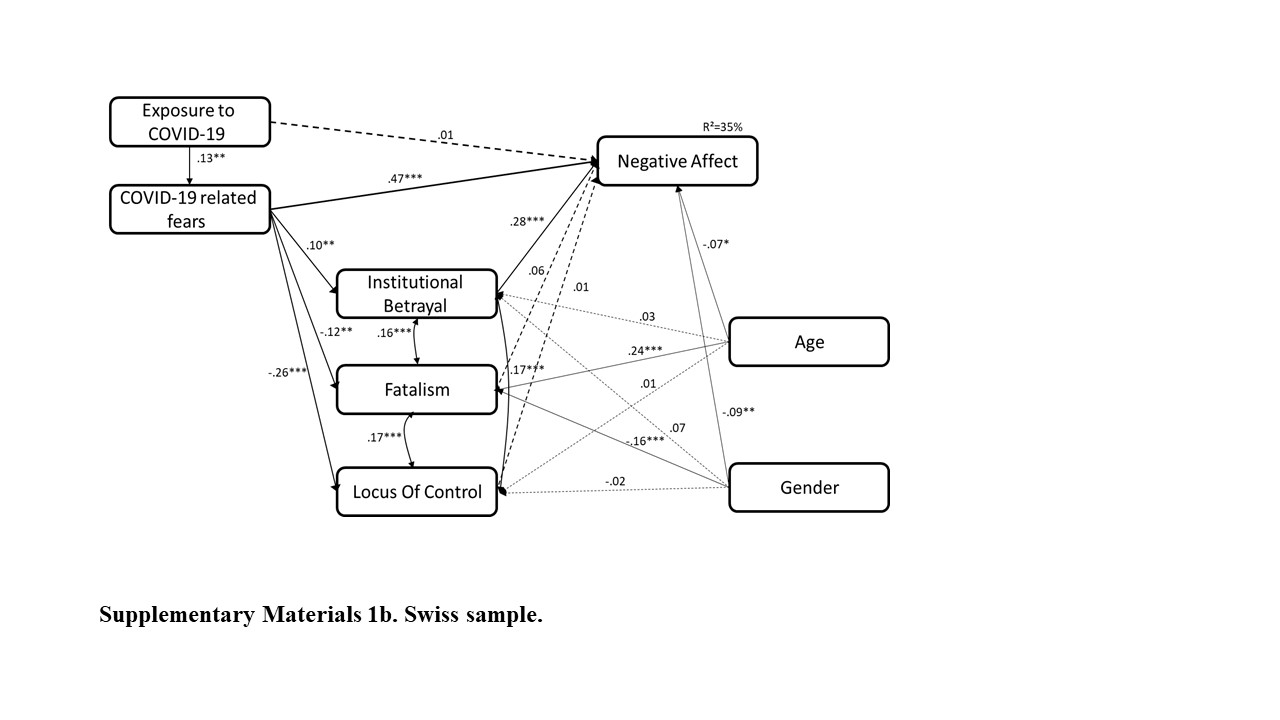

Supplement: Supplementary file 2 [file Image_2.JPEG]
